# Executive functions in motor imagery: support for the motor-cognitive model over the functional equivalence model

**DOI:** 10.1007/s00221-020-05756-4

**Published:** 2020-03-16

**Authors:** Scott Glover, Elys Bibby, Elsa Tuomi

**Affiliations:** Department of Psychology, Royal Holloway University of London, Egham, London, TW20 0EX Surrey UK

## Abstract

The motor-cognitive model holds that motor imagery relies on executive resources to a much greater extent than do overt actions. According to this view, engaging executive resources with an interference task during motor imagery or overt actions will lead to a greater lengthening of the time required to imagine a movement than to execute it physically. This model is in contrast to a currently popular view, the functional equivalence model, which holds that motor imagery and overt action use identical mental processes, and thus should be equally affected by task manipulations. The two competing frameworks were tested in three experiments that varied the amount and type of executive resources needed to perform an interference task concurrent with either an overt or imagined version of a grasping and placing action. In Experiment 1, performing a concurrent calculation task led to a greater lengthening of the time required to execute motor imagery than overt action relative to a control condition involving no interference task. Further, an increase in the number of responses used to index performance affected the timing of motor imagery but not overt actions. In Experiment 2, a low-load repetition task interfered with the timing of motor imagery, but less so than a high load calculation task; both tasks had much smaller effects on overt actions. In Experiment 3, a word generation task also interfered with motor imagery much more than with overt actions. The results of these experiments provide broad support for the motor-cognitive model over the functional equivalence model in showing that interfering with executive functions had a much greater impact on the timing of motor imagery than on overt actions. The possible roles of different executive processes in motor imagery are discussed.

## Introduction

Motor imagery represents a valuable tool in skill training (Blair et al. 1993; Denis 1985), and in rehabilitation following central or peripheral injury (Grabherr et al. 2015; Harris and Hebert 2015). Empirical investigations of motor imagery contribute greatly to understanding the internal mental representation underlying action production (Guillot et al. 2012; Moran et al. 2012; Rodriguez et al. 2008; Vogt et al. 2013). The traditional view of motor imagery holds that it operates using the same internal mechanisms as overt action (Decety 1996; Holmes and Collins 2001; Jeannerod 1994, 2001). However, whereas motor imagery often mimics the behavioural outcomes of overt actions (Decety and Michel 1989; Decety and Jeannerod 1995; Dixon and Glover 2009; Glover and Dixon 2013; Glover et al. 2004), numerous discrepancies have been observed in both the behavioural outputs (Calmels and Fournier 2001; Ceretelli et al. 2000; Coelho et al. 2012; Glover and Baran 2017; Slifkin 2008; Walsh and Rosenbaum 2009) and neural correlates (Gerardin et al. 2000; Hetu et al. 2013; Guillot et al. 2009; Nair et al. 2003) of imagined versus overt actions. Elucidating and accounting for the differences as well as the similarities between these two behaviours is of critical importance in the understanding of both.

The motor-cognitive model holds that the planning of both motor imagery and overt action rely on shared motor representations; during execution the two behaviours are subserved by different mental processes (Glover and Baran 2017). Whereas overt actions use visual and proprioceptive feedback to monitor and correct ongoing movements, these sources of information are unavailable to motor imagery due to the lack of physical movement. Instead, during execution motor imagery employs a conscious elaboration and monitoring process that draws heavily on executive resources. It is this functional bifurcation of motor imagery and overt actions during execution that leads to differences in their behavioural outputs under many circumstances. When an imagined action’s overt counterpart relies heavily on online control, or when the executive functions used during motor imagery are otherwise engaged, the performance of motor imagery is slowed relative to overt action (Glover and Baran 2017; Calmels et al. 2006).

A competing account of motor imagery, the functional equivalence model, holds that imagined movements and overt actions use the same mental processes (Decety 1996; Holmes and Collins 2001; Jeannerod 1994, 2001). According to this view, motor imagery consists of little more than the internal unfolding of a motor representation in the absence of physical movement. The functional equivalence view thus predicts a close match in both the behavioural characteristics and neural activation patterns of motor imagery and overt action.

Glover and Baran (2017) presented evidence for the motor-cognitive model and against the functional equivalence model. We showed that motor imagery was slowed more by tasks that emphasised online control, and by interference tasks that depleted executive resources, than was overt action. In one experiment, participants had to grasp and lift an egg that could be slippery or not slippery, or simply imagine doing so. Participants in both the overt action and motor imagery groups took longer to execute the task when the egg was slippery, requiring greater precision and a heavy reliance on online control, than when the egg was not slippery. Critically, this precision effect was much greater on motor imagery than on overt action. Similar interactions between motor imagery/overt action and high/low precision were also observed in experiments which involved grasping and placing a disc in either a small or large receptacle. These results supported the motor-cognitive model in that an increased reliance on online control affected motor imagery more than overt actions. The results were however inconsistent with the Functional Equivalence Model, which predicted matching effects of task manipulations on imagery and overt action.

Two further experiments by Glover and Baran (2017) tested whether interference with executive processes during execution would also impact motor imagery more than overt actions. Participants performed either an imagined grasping and placing task, or the corresponding overt action, while on some trials simultaneously performing a calculation task designed to tax executive resources, counting backwards by threes. Motor imagery was much slower when the imagined movement was executed simultaneously with the calculation task, whereas in the overt action condition the calculation task had only a minimal effect. These experiments further supported the motor-cognitive model over the functional equivalence model in suggesting that executive functions play a much greater role in motor imagery than in overt actions.

Other evidence for the motor-cognitive model comes from studies showing differences in the timing of overt and imagined actions (Calmels and Fournier 2001; Cerritelli et al. 2000; Decety et al. 1989; Slifkin 2008; Yoxon et al. 2015; Wong et al. 2013). For example, Slifkin (2008) had participants execute or imagine executing a simple finger movement while various weights were attached to their finger. Although low loads did not lead to any discrepancies between overt and imagined movement times, increasing the load tended to slow down motor imagery more than overt actions. Similar non-corresponding effects of an external load on overt and imagined movement times were reported by Decety et al. (1989). These mismatches between the timing of motor imagery and overt actions support the idea of a functional bifurcation between the two behaviours during execution.

Despite the findings of minimal effects of an interference task on overt actions observed by Glover and Baran (2017), there is evidence that overt actions can also be affected by interfering with executive functions (see Leone et al. 2017, for a review). For example, a classic study by Creem and Profitt (2001) demonstrated that a semantic interference task affected the choice of postures in grasping everyday tools. In another study, Friedman et al. (1988) found that having participants perform a concurrent verbal memory task slowed the performance of speeded tapping. Note, however, that apart from Glover and Baran (2017), none of the above studies directly compared overt actions with motor imagery. Given that we proposed a key difference between the Motor-Cognitive and Functional Equivalence Models relates to the role of executive functions in overt actions and motor imagery, respectively, it seems important to investigate the relative role of executive functions in imagery and action further.

The present set of three experiments were designed to extend the differential effects of a simultaneous calculation task on motor imagery and overt action observed by Glover and Baran (2017), by examining whether these effects generalise to other types of tasks involving executive functions. If the motor-cognitive model is correct, engaging executive functions with an interference task should slow motor imagery more than overt action regardless of the type of interference task used. Conversely, if the functional equivalence view is correct, all interference tasks should have similar effects on the timing of motor imagery and overt actions.

### Overview of the present study

All three experiments described here used the high precision grasping and placing motor imagery task from Glover and Baran (2017; Marteniuk et al. 1987), in which participants either executed, or imagined executing, reaching and grasping a disc and then placing it into a small cylinder. Each experiment varied the characteristics of the interference task to examine the effects of a variety of interference tasks on the timing of motor imagery and overt actions. Experiment 1 examined the effects of varying the number of times participants were required to press a key to index the completion of various stages of the overt or imagined movement on a given trial, and whether that task interacted with a calculation task. To anticipate, it was found that both increasing the number of keypresses and including the calculation task lengthened imagined movement times, whereas only the calculation task appeared to affect overt actions, and to an extent much less than its effects on motor imagery.

Experiment 2 compared the effects of the same calculation task used in Experiment 1 to a simpler task in which participants simply repeated the same number over and over. Both calculation and repetition affected motor imagery times, with the effects being larger for the high load calculation task than the low load repetition task. The interference effects on overt actions were again much smaller than their effects on motor imagery.

Finally, Experiment 3 had participants spontaneously generate words while simultaneously performing either the motor imagery or overt action. It was found that, as with the other interference tasks, word generation had a much larger impact on motor imagery times than on overt actions.

## Experiment 1

One possible way in which executive functions might be engaged during a typical motor imagery task is in switching attention between the monitoring and elaboration of the motor image and the responses used to index the beginning and completion of the imagined movement (Glover and Baran 2017). Switching attention between tasks is known to be a time-consuming process (Pashler 1998), which could at least partly explain why motor imagery often takes longer than an otherwise identical overt action (Calmels and Fournier 2001; Cerritelli et al. 2000; Decety et al. 1989; Glover and Baran 2017; Slifkin 2008; Wong et al. 2013; Yoxon et al. 2015). That is, if participants in the motor imagery version of the task are limited to performing either motor imagery or the interference task at any given time due to limitations in executive resources, then switching between these tasks might increase motor imagery times. Conversely, if participants in the overt action version of the task are able to perform both the action and the interference task simultaneously (i.e., if overt action uses minimal executive resources), then no costly task-switching would be required.

In Glover and Baran (2017), participants executed keypresses with their non-dominant hand to index the beginning and completion of the motor imagery or overt action. If switching attention between motor imagery and the keypresses slows the former down, then increasing the number of keypresses used to index various points of an imagined or overt movement ought to slow motor imagery even more, while having little effect on the timing of overt actions. If done in conjunction with another executive function task such as mental calculation, the effect of increasing the number of keypresses on motor imagery ought to be even larger.

The motor-cognitive model thus predicted that both increasing the number of indexing keypresses from two to three, and requiring participants to perform the calculation task, would affect motor imagery more than overt actions. If this is correct, then we ought to observe two separate interactions: first, an interaction between group, motor imagery (MI) vs. overt action (OA) and keypresses, whereby increasing the number of keypresses from two to three will have a greater effect on motor imagery than on overt actions. Second, an interaction between group and calculation whereby performing the calculation task ought to also have a greater effect on motor imagery than on overt actions. Conversely, the functional equivalence model holds that any task variables should affect both overt actions and motor imagery to the same extent. Thus, this model predicted no such interactions.

### Methods

#### Participants

Thirty-two participants from the Royal Holloway Department of Psychology undergraduate research participation pool took part in the study in return for course credit. All participants in this and the other two experiments provided their informed consent ahead of testing. One participant from the motor imagery group was removed from the analysis for failure to follow instructions. Participants were randomly assigned evenly into two groups of 16 that took part in either the motor imagery or overt action version of the task. This use of a between-subjects design ensured that participants in the motor imagery group could not use any overt experience of the task to determine the time required to imagine the movement (Pylyshn 2002). All participants were right-handed by self-report, had normal or corrected vision, had no motor or neurological impairments, and were naïve as to the exact purpose of the experiment. All participants spoke English as their first language. This and the other two experiments presented here were approved by the College Ethics Committee at Royal Holloway.

#### Apparatus and stimuli

**Fig. 1:**
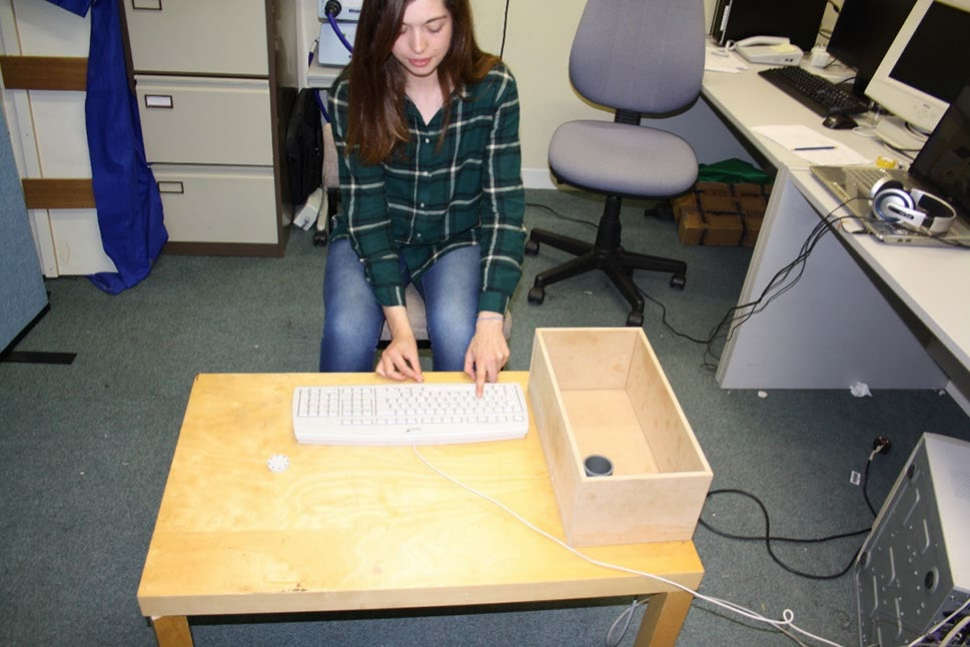
The experimental set up in Experiments 1, 2, and 3. The participant begins with their right hand in a pincer posture on the start mark and their left index finger resting on the ‘*d*’ key. On the sounding of a tone, participants either reach out to grasp the disc, then place it into the grey cylinder inside the box (overt action group), or simply imagine doing so (motor imagery group). The left hand in all cases presses the ‘*d*’ key to index different points in time of the overt or imagined movement

Participants sat at the long end of a 120 × 80 cm table on which was present a small disc, a box with a cylinder inside, and a computer keyboard (Fig. 1). The starting position was a circular pencil mark approximately 0.25 cm in diameter, drawn 8 cm from the edge of the table nearest to the participant, and halfway between each long end of the table. Participants sat so that the starting position was aligned with their body midline. The target disc was made of white plastic (4 cm in diameter and 1 cm thick), positioned with its centre 32 cm to the right and 10 cm forward of the starting position. To the left of the participant was situated a 15 cm high, 4.2 cm diameter solid grey plastic tube, centred 47 cm left and 40 cm forward of the starting position, placed inside of a 42 × 22 × 15.7 cm (height) wooden box, centred 56 cm to the left and 21 cm forward of the starting position. The box itself served no purpose in the experiment except to contain the cylinder. A computer keyboard was placed on the table in front of participants, positioned with its centre to the left of the participant’s midline such that the ‘*d*’ key was 15 cm to the left of, 9 cm in front of, and 2 cm above the starting position.

#### Procedure

Participants in both the overt action and motor imagery groups began each trial with their thumb and index finger held together in a pincer posture on the starting mark. Participants in the overt action group were instructed to execute a reaching and grasping action to lift the disc off the table using only their right thumb and index finger and then place it into the cylinder. Participants in the motor imagery group were instructed to imagine the same movement while keeping the right arm and hand motionless at all times. In the two keypress condition, participants had to press the ‘d’ key with their left index finger simultaneously with beginning the movement (or imagining the beginning of the movement in the motor imagery group), and then press it again simultaneously with releasing the disc into the cylinder (or imagining this same action). In the three keypress condition, participants had to press the ‘*d*’ key to indicate not only the beginning and end of the movement (or their imagined counterparts), but also the intermediate time at which they first grasped the disc (or imagined doing so). For the overt action condition, the experimenter monitored participants to ensure they pressed the key at the appropriate times.

Each trial began with the sounding of a tone by the experimenter. In the calculation condition, the experimenter first read each participant a randomly-generated number between 50 and 99, then immediately sounded a tone via the computer. In this condition, participants had to count backwards from the assigned number by threes while simultaneously performing the motor task (or its imagined equivalent). In the control condition, no number was given and the experimenter simply sounded the tone, at which time participants completed the task in silence.

Thus, participants in the motor imagery and overt action groups performed the exact same combination of tasks involving two or three keypresses with their left hand, and calculating backwards by threes on some trials. The only difference between the two groups was whether they overtly executed the grasping and placing task, or only imagined doing so. Participants in the motor imagery group were instructed to use both visual and kinesthetic imagery of their movement, experienced in the first person (i.e., to imagine seeing and feeling themselves performing the movement).

For each participant, half of the trials were two keypress trials and the other half were three keypress trials. Half of each of these were calculation trials and the other half were no calculation trials. Each of the four possible combinations of two/three indexing response and calculation/no calculation trials was presented 8 times in a blocked fashion for a total of 8 trials × 4 blocks = 32 trials, with the order of presentation of blocks counterbalanced across participants.

#### Design and analysis

The experiment used a 2 × 2 × 2 mixed design with Group as a between-subjects variable, and keypresses and calculation as within-subjects variables. Total movement times in both the motor imagery and overt action groups were measured as the time between the first and second keypresses for the two indexing response trials and the first and final keypresses for the three indexing response trials. For both types of trials, both overt and imagined movement times thus represented the total time between initiating and completing the entire imagined grasping and placing sequence. Mean movement times were analysed using nested linear models that included main effects of Group (motor imagery versus overt action), keypresses (two versus three), calculation (counting backwards by threes versus no calculation), and their interactions. The term “nested models” in this context refers to the fact that statistical models were sequentially compared to previous models wherein the latest model includes all the factors included in the next most recent model, plus one or more other variables.

To examine whether the manipulations affected the timing of the second vs. third keypresses differently, we ran a secondary analysis using a different 2 × 2 × 2 mixed design. Here, Group was again a between-subject variable, and calculation was a within-subjects variable. Here however, the third variable, Phase, was whether it was the second (grasping phase) or third (placing phase) keypress; this was again a within-subjects variable. Mean movement times were analysed using nested linear models as described above.

Following Glover and Dixon (2004), all analyses are reported using likelihood ratios; these provide an intuitive index of the strength of the evidence while avoiding many of the well-known pitfalls associated with the use of *p *values and NHST (Cohen 1994; Loftus 1996; Simmons et al. 2011; Wasserstein and Lazar 2016). As a model with more parameters will almost always fit the data better than a simpler model, adjusted likelihood ratios (*λ*_adj_) for each analysis were calculated based on the Akaike Information Criterion (AIC—Akaike 1973). In most prototypical hypothesis-testing scenarios, *λ*_adj_ = 3:1 corresponds to a *p *value ~ 0.05, whereas *λ*_adj_ = 10:1 corresponds to a *p* value ~ 0.02. Effects sizes are given as the *R*^2^ values associated with each model.

In accord with the principles of open science, the raw data and calculations used in the analyses for all three experiments are publicly accessible at the OSF website: https://osf.io/y9ak4/

### Results

**Fig. 2:**
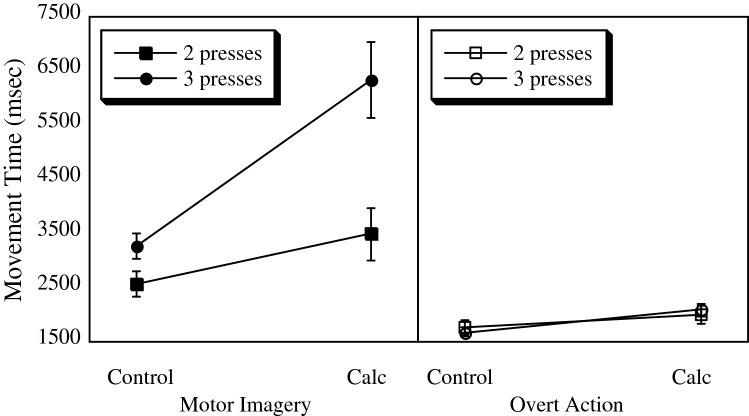
Effects of varying the number of indexing responses and the calculation task on movement times in the motor imagery (left panel) and overt action (right panel) groups. Calc, calculation task. Error bars represent standard errors of the means and are appropriate for between-group comparisons

The effects of the calculation task and the number of keypresses on motor imagery and overt action movement times on the remaining 31 participants are shown in Fig. 2. These data supported the hypothesis that increasing the number of keypresses would slow motor imagery. A model assuming an effect of Group (MI vs. OA) fit the data much better than did a null model, *λ*_adj_ > 1000, *R*^2^ = 0.65. This result showed the data were more than 1000 times as likely given an effect of Group on movement times than given no such effect. Adding in the main effects of keypresses and calculation improved the fit further, *λ*_adj_ > 1000, *R*^2^ = 0.27.

As predicted by the Motor-cognitive model, there was strong evidence that a model including the Group × keypresses and Group × calculation interactions fit the data better than a model including only the main effects, *λ*_adj_ > 1000, *R*^2^ = 0.45. The effect of the calculation task was much larger for the motor imagery group (mean difference between calculation and no calculation 2217 ms) than for the overt action group (mean difference 324 ms). Further, the effect of increasing the number of keypresses from two to three was only evident in the motor imagery task (1548 ms difference for motor imagery versus 7 ms difference for the overt action group). Finally, a full model including the above-mentioned effects, as well as the keypresses × calculation interaction and the three-way interaction fit the data somewhat better than a model excluding the keypress × calculation and three-way interactions, *λ*_adj_ = 6.4, *R*^2^ = 0.49.

The secondary analysis involving the breakdown of results by phase. Movement times for the second (grasping phase) and third (placing phase) keypress are shown in Table 1. A model including an effect of Group fit the data better than a null model, *λ*_adj_ > 1000, *R*^2^ = 0.58, with movement times being longer in the motor imagery than overt action conditions. A model that included effects of calculation and its interaction with Group fit the data much better than a null model, *λ*_adj_ > 1000, *R*^2^ = 0.52, with movement times slowing more for motor imagery than for overt action when participants had to perform the calculation task. This latter model was also modestly favoured over a full model that included the above-mentioned effects, and added the main effect of phase and all other interactions, *λ*_adj_ > 5.1, *R*^2^ (of full model) = 0.53.

**Table 1:** Movement times as a function of grasping and placing phases in Experiment 1 (standard errors in parentheses)

Group	Calc. condition	Grasp phase MT	Place phase MT
Motor imagery	None	1593 (161)	1698 (111)
	Calculation	3000 (436)	3337 (376)
Overt action	None	816 (36)	893 (45)
	Calculation	1052 (61)	1055 (62)

### Discussion

The results provided further support for the motor-cognitive model of motor imagery over the functional equivalence model. The critical interactions between Group × keypresses and Group × calculation were both as predicted by the motor-cognitive model, and were in accord with its argument that the switching of attention between the motor imagery and indexing responses results in the lengthening of imagined movement times; no such effect was apparent in the overt action group. The calculation task also had a large effect on motor imagery compared to a much smaller effect on overt actions. This further engagement of executive resources impacted motor imagery much more than overt actions. These results suggested that the keypresses represent a second source of interference that contributes to the lengthening of motor imagery times relative to overt actions.

Although the predictions of the motor-cognitive model found clear support in Experiment 1, it is not obvious whether the switching of attention back and forth between motor imagery and the interference tasks was the sole source of the effects, or whether the calculation task might also impact motor imagery through the use of executive resources for the mental aspects of calculation. On the one hand, it may be simply that the switching of attention between calculation and motor imagery slows the latter down, in which case the type of interference task used ought to be irrelevant. On the other hand, it may be that the high cognitive load of the calculation task also impairs motor imagery by depleting executive resources needed to elaborate and monitor the motor image. These two possibilities were tested in the next experiment.

## Experiment 2

The nature of the interference effects on motor imagery and overt action was tested further using two versions of an interference task that varied in terms of cognitive load. Participants performed either overt actions or motor imagery in one of three conditions: (1) a calculation task identical to that used in Experiment 1, representing the high load version of the interference task; (2) a low load repetition task in which participants simply repeated the number given to them; or (3) a control condition in which participants simply executed the action (or its imagined equivalent) in silence. To quantify the level of cognitive load in the interference task, we also recorded the number of responses provided as a function of Group and calculation conditions.

If, as the motor-cognitive model of motor imagery posits, executive resources are more heavily engaged during motor imagery than during overt actions, then imagined movement times should be the longest when performed simultaneously with the high load calculation task, should be shorter in the low load repetition task, and shortest of all in the no load control condition. Conversely, as was observed in Experiment 1, any effects of the interference task should be minimal when the action was performed overtly. However, if the functional equivalence model is correct, and both overt actions and motor imagery utilise the same mental resources, there should be equivalent effects of the interference task on the two behaviours.

### Methods

#### Participants

Thirty-two new participants from the research participation pool in the Department of Psychology, Royal Holloway, took part in exchange for course credit. Participants were randomly split into equal cohorts of sixteen in the motor imagery and overt action groups. All were right-handed by self-report, had normal or corrected vision, had no motor or neurological impairments, and were naïve as to the exact purpose of the experiment. All participants spoke English as their first language.

#### Stimuli and apparatus

The apparatus was the same as in Experiment 1. Stimuli were two sets of 12 numbers from 50–99. For each participant, each set of numbers was assigned to either the calculation or repetition condition, with the assignment of sets to conditions counterbalanced across participants.

#### Procedure

As in Experiment 1, participants in both the overt action and motor imagery groups began each trial as before, with the thumb and index finger of their right hand held in a pinch on the starting mark, and their left index finger resting on the ‘*d*’ key of the keyboard. Again, participants either performed, or imagined performing, the same grasping and placing task as in Experiment 1. Participants in the motor imagery group were again instructed to imagine the action both visually and kinesthetically from the first-person perspective while keeping their right hand motionless.

Both groups took part in three conditions, presented in blocks, with the order of presentation counterbalanced across participants. In the calculation and repetition conditions, participants were read a number from 50–99 prior to the sounding of a tone by the experimenter via the computer. The calculation task was performed as in Experiment 1—i.e., participants were required to count backwards by threes from the number read to them while performing either the overt action or motor imagery grasping and placing task. In the repetition condition, participants had to only repeat the number that was read to them at a pace of one repetition per second (or as close to it as possible) while simultaneously performing the motor imagery or overt action task. In the control condition, the experimenter simply sounded the tone when the participant was ready, after which the participant imagined or overtly executed the movement in silence. In all conditions, at the sounding of the tone participants pressed the button once with their left index finger when they began (or imagined beginning, for the motor imagery group) the movement, and a second time when they released the disc into the cylinder (or imagined doing so). The experimenter recorded the number of items generated in both the repetition and calculation conditions; errors in calculation were ignored as the interest here was in the number of responses generated rather than the accuracy of the calculations being performed. Participants executed 12 trials in each of the three blocks, for a total of 12 trials × 3 blocks = 36 trials.

#### Design and analysis

Movement time analysis used a mixed 2 × 3 design, with two levels of the between-subjects variable Group (MI vs. OA) and three levels of the within-subjects variable Interference (control, repetition, calculation). Imagined and overt movement times were measured as the time between the first and second keypress responses. Number of responses were analysed using a mixed 2 × 2 design, with the same two levels of the between-subjects variable Group as above, but only two levels of the interference variable (repetition and calculation), as no responses were required in the control condition. As before, the fit of nested linear models were compared using adjusted likelihood ratios, with effect sizes reported as *R*^2^ values.

### Results

**Fig. 3:**
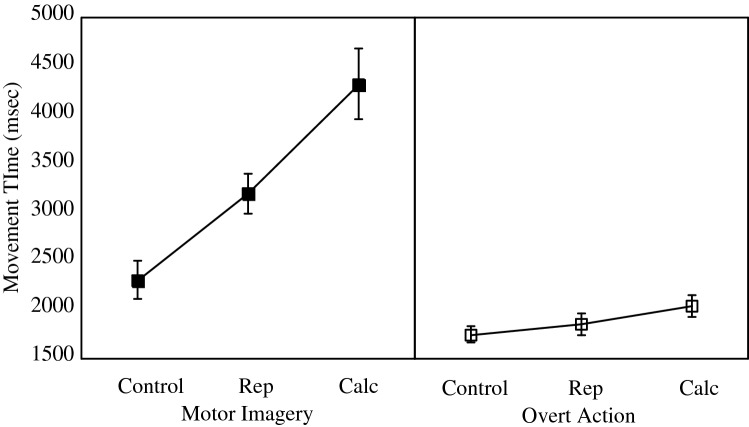
Effects of the repetition (Rep) and calculation (Calc) tasks on movement times in the motor imagery (left panel) and overt action (right panel) groups. Error bars represent standard errors of the means and are appropriate for between-group comparisons

Figure 3 shows the effects of the different interference conditions on movement times in the motor imagery (left panel) and overt action (right panel) groups. A model that included an effect of Group on movement times fit the data better than a null model, *λ*_adj_ > 1000, *R*^2^ = 0.46, meaning the data were more than 1000 times as likely given an effect of Group than given no such effect. Adding in an effect of calculation improved the fit further, *λ*_adj_ > 1000, *R*^2^ = 0.49.

Critically, as predicted by the motor-cognitive but not the functional equivalence view, the best fit was achieved by a full model that also included the interaction between Group × calculation, *λ*_adj_ > 1000, *R*^2^ = 0.76. Relative to the control condition, repetition increased movement times by a mean of 883 ms in the motor imagery group versus 97 ms for the overt action group, whereas for calculation relative to control conditions the effects on motor imagery and overt action were 2012 ms and 283 ms, respectively.

Table 2 shows the number of correct answers given in each combination of Group and calculation condition for which responses were required. As with movement times, the best-fitting model included main effects of both variables, and their interaction. A model including an effect of Group fit the data better than a null model, *λ*_adj_ > 1000, *R*^2^ = 0.59, with more responses occurring in the motor imagery than overt action condition. Adding in an effect of calculation condition improved the fit further, *λ*_adj_ > 1000, *R*^2^ = 0.64. Finally, a full model incorporating both main effects and the interaction fit the data better than a model based only on the main effects, *λ*_adj_ > 1000, *R*^2^ = 0.83. These results were as expected: more responses occurred in the repetition condition in which the cognitive load was low, and the effect of condition was greater in the motor imagery than overt action group.

**Table 2:** Mean number of correct responses given in each combination of Group and calculation conditions (standard errors in parentheses)

Group	Calc. condition	Number of responses
Motor imagery	Repetition	3.31 (0.23)
	Calculation	1.90 (0.19)
Overt action	Repetition	1.35 (0.13)
	Calculation	0.94 (0.10)

### Discussion

The results of Experiment 2 again supported the predictions of the motor-cognitive model. First, the effects of the interference tasks were much greater on motor imagery than on overt actions. Second, the interference effect was greater when participants had to perform the high load calculation task than the low load repetition task, supporting the notion that depleting executive resources impacts motor imagery to a much greater extent than it does an otherwise identical overt action task.

Given that even simple repetition of the same number manifestly slowed motor imagery relative to the control condition, the result also converges with that of the number-of-keypresses manipulation in Experiment 1 in showing that switching attention between motor imagery and an interference task can lengthen imagined movement times, even when that task itself entails only a nominal cognitive load. The larger effects of the calculation condition on imagined movement times, despite fewer responses being given in a longer period of time, suggests that the higher cognitive load involved in calculating had an independent effect on the timing of motor imagery above and beyond the switching of attention effect arising in the repetition condition.

Although the results of Experiment 2 were consistent with the view that the use of executive resources during the high cognitive load task interfered with the execution of the motor imagery task independent of the switching of attention between tasks, it is important to examine whether the interference effects generalise to other tasks that employ executive functions. If motor imagery indeed uses executive functions during execution, then the precise nature of the interference task should not matter as long as it has a significant cognitive load. Further to this, as in the Experiment 2, a higher load task should lengthen motor imagery times more than a comparable task with a lower load. Conversely, if the effects of the calculation/repetition on motor imagery did not generalise to another interference task, this would cast doubt on the idea that executive functions play a role in motor imagery.

## Experiment 3

The different theoretical possibilities outlined above were tested using putatively easy or hard versions of a word generation task performed simultaneously with the motor imagery or overt action tasks. Here, participants were presented with a random consonant prior to each trial and had to generate words out loud while simultaneously executing either the same motor imagery or overt action tasks as in Experiments 1 and 2. In the easy version of the word generation task, participants were allowed to generate words of any length, whereas in the hard version, participants had to generate words of specifically four letters in length. In the control condition, no letter was presented and the motor imagery or overt action was performed in silence. If executive functions are used much more during the execution of motor imagery than overt actions, as posited by the motor-cognitive model, then the word generation task ought to impact motor imagery to a greater extent than overt actions. Specifically, we ought to see the same Group x interference interaction as before. More generally, if executive functions are utilised by motor imagery processes, then changing the interference task from calculating (or repeating) numbers to generating words ought not to alter the overall nature of its effect on motor imagery.

The two models thus made the same predictions as before: The motor-cognitive model predicted an interaction in which the word generation task would have a greater effect on motor imagery than on overt actions. Conversely, the functional equivalence model predicted no such interaction. As in Experiment 2, we again recorded the number of responses provided in to quantify the difficulty of the two versions of the word generation task.

### Methods

#### Participants

Thirty-two new participants from the undergraduate research participation pool in the Department of Psychology, Royal Holloway took part in the study in return for course credit. As before, sixteen participants were randomly assigned to each of the motor imagery and overt action groups. All participants were right-handed by self-report, had normal or corrected vision, had no motor or neurological impairments, and were naïve as to the exact purpose of the experiment. All participants spoke English as their first language.

#### Apparatus and stimuli

The same apparatus was used as in Experiment 1. The stimuli now included a list of sixteen randomly-generated consonants presented to the participants, divided randomly into two sets of eight, and excluding J, V, Q, X, Y, and Z. For each participant, each set of eight consonants was assigned to either the hard generation or easy generation condition. The assignment of the consonants to condition was counterbalanced across participants.

#### Procedure

Participants began each trial as in Experiments 1 and 2. As before, the task only differed between groups in terms of whether they overtly executed, or imagined executing, the grasping and placing of the disc in the cylinder. Again, participants in the motor imagery group were instructed to imagine the movement both visually and kinesthetically from the first person while keeping their right hand and arm motionless.

The easy and hard word generation conditions and control trials were each presented as blocks with order of presentation counterbalanced across participants. In both word generation conditions, the experimenter began each trial by reading a consonant from the list out to participants and then immediately sounding the tone. Participants had to generate words out loud of either any length (easy) or four letters long (hard), while simultaneously performing the grasping and placing movement (or imagining it for the motor imagery group). As before, both groups indicated the start and end of the movement (or imagined movement) by pressing the ‘*d*’ key. In the control condition, no letters were presented, and participants performed the overt action or motor imagery task in silence. The experimenter recorded the number of words generated on each trial.

#### Design and analysis

The analysis of movement times used a 2 × 3 mixed design, with Group (MI vs. OA) as the between-subjects variable, and word generation (control, any length, four letters) as the within-subjects variable. The mean number of words and the mean movement time for each participant in each group and in each condition were analysed using adjusted likelihood ratios as before. The analysis of number of correct responses used a 2 × 2 mixed design, with the same two levels of Group, but only two levels of word generation (any length versus four letters), as no responses were required in the control condition.

### Results

**Fig. 4:**
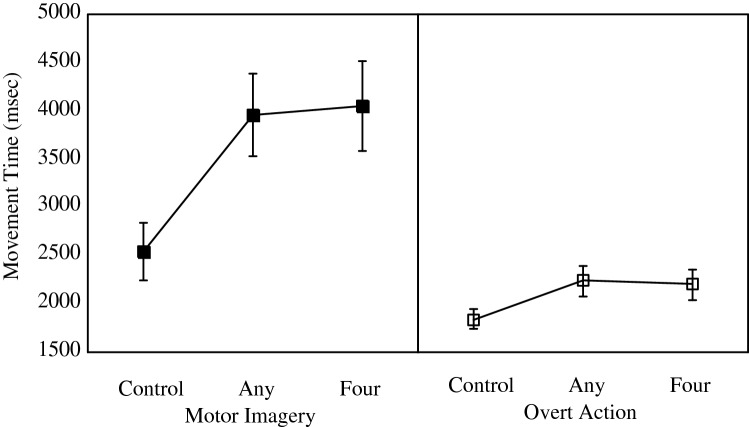
Effects of the word generation task on movement times in the motor imagery (left) and overt action (right) groups, compared to the control condition. Any: words generated could be of any length. Four: words generated had to be four letters long. Error bars represent standard errors of the mean and are appropriate for between-group comparisons

Figure 4 shows the mean movement times in the control, any length, and four letter word generation conditions, for motor imagery (left panel) and overt action (right panel). A model incorporating an overall effect of Group fit the data better than a null model, *λ*_adj_ = 190.4, *R*^2^ = 0.32. Adding in an effect of word generation condition improved the fit further, *λ*_adj_ > 1000, *R*^2^ = 0.33.

Critically, and in line with the motor-cognitive Model, the data showed clearly that there was an effect of word generation that was greater in the motor imagery than overt action condition, although it differed little between the any-length and four-letter conditions. Adding in the Group × word interaction improved the fit further still, *λ*_adj_ = 53.0, *R*^2^ = 0.44, showing the data were 53 times as likely assuming the Group × word interaction was present than that it was not. The mean difference between the control and any-length word generation condition was 1421 ms versus 397 ms in the motor imagery and overt action groups, respectively; the mean differences between the control and four-letter word generation conditions for motor imagery and overt action were 1507 ms and 356 ms, respectively.

The number of words generated in each condition and group are given in Table 3. As in Experiment 2, the pattern of results suggested the manipulation of task difficulty in the word generation conditions was successful in terms of the number of responses generated. A model incorporating a main effect of Group fit the data better than a null model, *λ*_adj_ = 1000, *R*^2^ = 0.66, indicating that more words were generated in the motor imagery group overall than in the overt action group. Adding in an effect of word generation improved the fit further, *λ*_adj_ > 1000, *R*^2^ = 0.48, and the fit was improved further by a full model that included the interaction, *λ*_adj_ = 1000, *R*^2^ = 0.67.

**Table 3:** Number of words generated in each combination of Group and word generation condition in Experiment 3 (standard errors in parentheses)

Group	Word generation	Number of words
Motor imagery	Any length	3.80 (0.23)
	Four letters	2.60 (0.26)
Overt action	Any length	1.39 (0.17)
	Four letters	1.11 (0.09)

## Discussion

Experiment 3 was successful in further generalising the larger interference effects on motor imagery than on overt action to a new task. The larger effects of the word generation task on motor imagery versus overt actions was consistent with the view that motor imagery is much more dependent on executive resources than is overt action.

The data also suggested that manipulating the difficulty of the word generation task did not modulate movement times much, if at all, in either the motor imagery or overt action group. This was not as predicted. However, it was clear that the participants were able to produce more words in the putatively easier condition in which words of any length were allowed, as opposed to when only four letter words were allowed, and this was especially evident in the motor imagery condition in which overall more words were generated. A possible explanation for this assumes participants switched back and forth between word generation and motor imagery in that condition, but were better able to perform the task simultaneously in the overt action condition.

On this analysis, the similar effects of the four-letter and any-length condition on movement time in the motor imagery condition would depend on the summing of the effects of switching attention and word generation. Specifically, on average fewer switches of attention took place in the four-letter than any-length condition, as fewer words were generated. In contrast, the time spent generating each word may have taken longer in the four-letter than any-length condition; this would be consistent with the expectation that it is harder to generate words of a specific length versus words of any length.

The cumulative effect of these two factors of switching attention and generating words may have been to cancel one another out: In the four-letter condition, participants spent relatively *more* time attempting to generate the next word, but because they produced fewer words as a consequence, relatively *less* time switching attention between word generation and motor imagery. Conversely, in the any-length condition, participants spent relatively *l*ess time generating each word, but because this led to more words being generated, they spent relatively more time switching attention between word generation and motor imagery. Notably, however, such a pattern did not arise in Experiment 2, in which the low load repetition condition had shorter movement times as well as more responses than the high load calculation condition. A difference may be that the any-length condition of Experiment 3 was more difficult than the low load repetition condition of Experiment 2. Certainly its effects on motor imagery times were greater (1421 ms vs. 883 ms longer movement times in motor imagery for the any-length [Experiment 3] and repetition [Experiment 2] conditions, versus the control conditions in the respective experiments.

In sum, the results of Experiment 3 conform with those of Experiment 1 and 2 in showing that concurrent performance of an interference task had a much larger effect on the timing of motor imagery than on a corresponding overt action. This is again consistent with the predictions of the motor-cognitive model but not the functional equivalence model.

## General discussion

The three experiments here sought to test the predictions of the motor-cognitive model against those of the functional equivalence view, using several tasks designed to engage executive functions while participants concurrently performed motor imagery or overt action. Each experiment provided valuable information in this regard, and taken as a whole they support the notion that executive functions are used during the execution of motor imagery to a much greater extent than during overt actions. These findings are important in showing that motor imagery is not functionally equivalent to overt actions as has sometimes been argued (Decety 1996; Holmes and Collins 2001; Jeannerod 1994, 2001), but rather that motor imagery has its own form and organisation that differs in at least some respects from overt action, as argued by the motor-cognitive model (Glover and Baran 2017). Whereas we contend that motor imagery most likely does use the same motor representations as overt actions during the planning stage, in the light of the current findings, we find it difficult to accept the functional equivalence argument that motor imagery is little more than the unfolding of these representations with the physical aspects of the movement suppressed.

The experiments reported here demonstrated that a variety of factors associated with executive functions interfered with the timing of motor imagery, and each experiment provided possible insights into the specific executive processes involved. Experiment 1 showed that increasing the number of keypresses used to index the timing of different components of the movement affected only motor imagery and not overt actions, while performing a calculation task had much larger effects on the timing of motor imagery than on overt actions. These results replicated and extended the findings of Glover and Baran (2017) that motor imagery was more affected by a calculation task that engages executive resources than was overt action.

Experiment 2 added to this by showing that a repetition task with a low cognitive load interfered with the timing of motor imagery, but to a lesser extent than a high load calculation task. Nonetheless, the effects of even this low load task on motor imagery were still several times higher than the comparable effects on the timing of overt action. The effects of the low load repetition task suggested, in line with the effects of increasing the number of keypresses in Experiment 1, that the switching of attention between motor imagery and an interference task is a key element in slowing motor imagery movement times. Further, the larger effects in the calculation than repetition condition suggest that the depletion of executive resources by a high cognitive load calculation task also played a significant role in the interference effects, above and beyond the switching of attention likely contributing to the effects of the low load repetition task. That is, in the high load calculation task, participants not only had to switch attention between tasks, but also had to perform a demanding mental operation. However, in the low load repetition task, the main use of executive functions would have been to switch between it and the motor task (albeit with some monitoring of responses necessary given the instruction to maintain a pace of approximately one repetition/second).

Experiment 3 demonstrated that the interference effects generalised to a different type of executive function task, word generation. Although the presumably harder version of the task which required words of four letters length be generated did not interfere more with motor imagery than the easy version of the task in which words of any length could be generated, this result might have been confounded by the greater number of responses in the latter task, which may have involved more frequent switching of attention. A future study may clarify this by providing levels of the task that more clearly differ in their cognitive load.

A champion of the functional equivalence view might attempt to explain the results of the present study by arguing that the interference tasks in all three experiments did indeed affect the timing of both motor imagery and overt actions. They might posit that the results arose because participants in the motor imagery group simply engaged with the interference task more than did those in the overt action group, giving rise to an artefact. We find this explanation unlikely, however, for the following reasons: First, instructions to the motor imagery and overt action groups did not differ apart from whether they were performing motor imagery or overt action, making it difficult to explain why one group should consistently prioritise performance of the interference tasks whereas the other did not. Second, the longer movement times in the interference conditions for the motor imagery group also occurred when the interference task involved only a nominal cognitive load, such as the keypressing manipulation of Experiment 1 and the repetition task of Experiment 2. Overall, we find our explanation of these results more plausible. Specifically, we hold that participants were unable to perform motor imagery and the interference tasks simultaneously, owing to both relying on a limited pool of executive resources. This required participants to switch attention between the motor imagery and interference tasks (a prediction of the motor-cognitive model). Conversely, participants were able to perform the overt action and interference tasks simultaneously, resulting in relatively minimal effects of the interference tasks on movement times in the overt action condition.

More generally, the results of all three experiments are difficult to reconcile with a functional equivalence view which predicts comparable effects of task variables on motor imagery and overt action. For this view to have been upheld, none of the interactions between motor imagery/overt action and the interference tasks in the three experiments should have been observed. The motor-cognitive model thus appears to give a superior account of the results of the present study.

Although the motor-cognitive model outperforms the functional equivalence view in explaining the present results, other explanations can also be offered. For example, it may be argued that the mental process of motor imagery is simply more easily distracted than overt actions, as only the latter receives ongoing proprioceptive feedback. Similarly, it may be that participants are more inclined to focus on the secondary task at the expense of motor imagery relative to overt action. Although these views are plausible, our sense is that a much greater use of executive functions by motor imagery than overt actions provides a more parsimonious account of the present results. Specifically, we note that the interference tasks were not strictly “distracting” in the sense that the imagery/action task was the sole priority and the interference task merely diverted attention from it. Rather, participants were instructed to perform both the imagery/action task and the secondary task simultaneously; they simply had much more difficulty performing motor imagery than overt action in this situation, as evidenced by the slower movement times in the former as compared to the latter. That said, our results cannot fairly rule out the above explanation that participants prioritised the secondary task when performing motor imagery than overt actions.

An interesting prediction of the motor-cognitive model that could address the above argument regarding prioritisation is that interference effects should be bi-directional. That is, if executive resources are shared between motor imagery and an interference task, then not only should the interference task slow motor imagery, but motor imagery should slow performance of the interference task as well. This would be consistent with other results showing that an intervening task can impair the recall of items in a working memory task (Nieuwenstein and Wyble 2014), presumably through taking command of executive resources needed for rehearsal (Baddeley and Hitch 1974). Bi-directional effects would further support the idea of a sharing of executive resources between motor imagery and an interference task.

Several other questions of interest remain following the present study. One is the exact nature of the process when an interference task is performed simultaneously with motor imagery. Are both tasks performed concurrently, but at a slower rate, or must the tasks be completed in separate stages, due to a resource bottleneck (Pashler 1998)? Introspectively, although it seems to us possible to perform motor imagery concurrently with another cognitively demanding mental task, it also seems easier to separate the tasks mentally and to switch back and forth between them, than it does to attempt to do them simultaneously. It thus seems plausible to suggest that among the various interference tasks used here, the large effects on the timing of motor imagery resulted because participants divided their trial periods up between the interference tasks and the motor imagery, resulting in fewer responses for more difficult interference tasks (where each response took more time to generate) and more responses for easier interference tasks (where each response took less time to generate).

There are several putative roles of executive functions in cognition beyond the switching of attention (e.g., Rogers and Monsell 1995) and performance of mental operations (e.g., Gilbert and Burgess 2008) so far considered here. For example, the maintenance of items in working memory (e.g., Baddeley and Hitch 1974), and response inhibition (Logan 1983) could both arguably have played a role in the interference effects we observed on motor imagery. It may be, for example, that holding an item in working memory interferes with the elaboration of a motor image.

It might also be argued that the inhibition of physical movement during motor imagery is disrupted by the concomitant use of executive functions to perform an interference task, leading to an overall slowing of motor imagery (Rieger et al. 2017). This view is somewhat consistent with data showing that areas of the brain involved in response inhibition are more active during motor imagery than overt actions, including the SMA (Hetu et al. 2013). Our sense, however, is that such low level motoric response inhibition is unlikely to result in the large effects of the interference tasks observed here. Indeed, if the requirement to inhibit overt responses were responsible for a slowing of motor imagery, then motor imagery ought to always take longer than overt actions. This, however, is far from the case (Guillot and Collet 2005). Further, a study that specifically examined the crossover of inhibition effects between motor imagery and overt actions reported effects only in the tens of milliseconds (Rieger et al. 2017), rather than in the hundreds of milliseconds observed in the present study. Instead, our view is that the bulk of the disruption occurs at the higher cognitive level of executive functions, through processes involved in switching attention and performing mental operations. We suggest that these processes are identifiable with the dorsolateral prefrontal cortex (DLPFC). Concordant with this view, studies show greater activity in DLPFC during motor imagery than overt actions (Guillot et al. 2009), that this tendency increases for less familiar actions (Zhang et al. 2018), and that motor imagery training can increase the responsiveness of DLPFC (Moriva and Sakatani 2017). The suggested role of executive functions in motor imagery is also consistent with the putative critical role of the DLPFC in executive functions (Niendam et al. 2012; Yuan and Raz 2014).

## Conclusion

The present study tested the effects of various interference tasks on the timing of motor imagery and overt action. In all cases, the interference tasks affected the timing of motor imagery much more than of an otherwise identical overt action. These effects appeared to be due to the engagement of executive resources by the interference task, either through the need to switch attention between the interference task and motor imagery, or through the mental operations required by the interference task itself (or some combination of both). These results provide strong support for the motor-cognitive model, in which motor imagery uses internal motor representations in planning, but relies on executive resources to monitor and elaborate the image during execution. The results were at odds with the functional equivalence model, however, in which motor imagery and overt actions are held to rely on the same mental processes. In light of these findings, we believe the motor-cognitive model represents a promising framework for understanding motor imagery and its relation to overt action.
